# Sodium Bicarbonate in Gastric Diseases

**Published:** 1913-11

**Authors:** 


Sodium Bicarbonate in Gastric Diseases. Dr. E. Binet, Vichy. Only moderate doses of bicarbonate of soda must be given; the dose of one drachm and a half is the maximum daily dose. Under such conditions the patients to whom this salt must be prescribed are those affected with disorders of gastric evacuation, and whose stomach empties itself too slowly. Late evacuations are met with in two cases and characterize two conditions. In the first case, owing to insufficient peristaltic contractions, the churning of the alimentary mass is too slow and insufficiently stimulates the opening of the pylorus. In the second case, the muscle has retained its normal tonus; but the contractions which it causes, however strong they may be, are only able to overcome very slowly the spasm which keeps the pylorus closed. In the first condition the diminution of the secretion, hypopepsia, is parallel with the muscular atony; it is the condition of its evolution, and its very degree enables us to evaluate the degree of the gastric hypotonicity. In the other condition on the other hand, hyperpepsia is generally present and the higher its percentage, the easier and the stronger is the reflex conclusion of the pylorus.

Clinically gastric pain in itself seems to be a sign of abnormal evacuation, whatever be its form in the course of digestion or its conditions of time and duration.

For all these reasons bicarbonate of soda is indicated in a great many cases. Except in cases when there is an acute ulceration (haematemesis or melaena) and except in some cases of gastric cancer, its use may be freely recommended. It must be considered not so much as giving an immediate and temporary result, but as a regulator of gastric digestion. Therefore, it must be given to prevent pain rather than to stop pain. This is why it seems rational to prescribe it in small doses repeated in the course of the same digestion.

The following combination seems to be the best to prescribe:

| | |
|---|---|
| Sod. Bicarb | 0.75 |
| Magn. Pond | 0.25 |
| Pulv. Bellad. Fol | 0.01 |

Patients with dyspeptic pains connected with motor insufficiency must take two of these powders an hour and half an hour before meals, and even half an hour and an hour after the same meal. Patients whose delayed evacuation is connected with a pyloric spasm, produced or kept up by hypersecretion, take these powders during the whole of their digestion, beginning an hour after the meal and continuing at intervals of an hour and a half until the next meal.

This modus operandi is to be preferred to Mathieu's treatment. Mathieu gives a teaspoonful of

| | |
|---|---|
| Sod. Bicarb | oz.ss. |
| Magn. Pond | dr. j |

Bourget's mixture may also be prescribed; it is a solution of dr. ij of sod. bicarb., dr. ss Sod. phosph, exsic. dr.ss, and sod. sulph. dr. ss., in 1000 c.c. of water; 150 to 200 c.c. to be taken first thing in the morning as soon as the pain appears.

It may be advisable to bring the solution of sod. bicarb to about 100° F. since it is well known that fluids the temperature of which closely approaches body heat are less irritating for the mucous membranes to which they come in contact.

In spite of all this and in spite of all the advantages of the alkaline treatment with bicarbonate of soda, it cannot be expected to work wonders even in the most suitable cases. A suitable dietetic treatment must by all means be prescribed at the same time. Even if this mode of treatment has to be prolonged as long as the pains persist, it is useless to reduce the dose as soon as the pains are less severe or less frequent; in other words, this treatment must be started at the same time as the dietetic treatment, but it must in no case be prolonged after the dietetic treatment has been stopped.

